# Redefining postoperative hypertension management in carotid surgery: a comprehensive analysis of blood pressure homeostasis and hyperperfusion syndrome in unilateral vs. bilateral carotid surgeries and implications for clinical practice

**DOI:** 10.3389/fsurg.2024.1361963

**Published:** 2024-04-04

**Authors:** Sherif Sultan, Yogesh Acharya, Makinder Dulai, Wael Tawfick, Niamh Hynes, William Wijns, Osama Soliman

**Affiliations:** ^1^ Department of Vascular and Endovascular Surgery, Western Vascular Institute, University Hospital Galway, University of Galway, Galway, Ireland; ^2^ Department of Vascular Surgery and Endovascular Surgery, Galway Clinic, Royal College of Surgeons in Ireland and University of Galway, Galway Affiliated Hospital, Doughiska, Ireland; ^3^ CORRIB-CURAM-Vascular Group, University of Galway, Galway, Ireland; ^4^ The Euro Heart Foundation, Amsterdam, Netherlands

**Keywords:** carotid artery stenosis, carotid endarterectomy, hypertension, hyperperfusion syndrome, stroke

## Abstract

**Background:**

This study evaluates the implications of blood pressure homeostasis in bilateral vs. unilateral carotid surgeries, focusing on the incidence of postoperative hypertension, hyperperfusion syndrome, and stroke as primary outcomes. It further delves into the secondary outcomes encompassing major adverse cardiovascular events and all-cause mortality.

**Methods:**

Spanning two decades (2002-2023), this comprehensive retrospective research encompasses 15,369 carotid referrals, out of which 1,230 underwent carotid interventions. A subset of 690 patients received open carotid procedures, with a 10-year follow-up, comprising 599 unilateral and 91 bilateral surgeries. The Society for Vascular Surgery Carotid Reporting Standards underpin our methodological approach for data collection. Both univariate and multivariate analyses were utilized to identify factors associated with postoperative hypertension using the Statistical Package for the Social Sciences (SPSS) Version 22 (SPSS®, IBM® Corp., Armonk, N.Y., USA).

**Results:**

A marked acute elevation in blood pressure was observed in patients undergoing both unilateral and bilateral carotid surgeries (*p* < 0.001). Smoking (OR: 1.183, *p* = 0.007), hyperfibrinogenemia (OR: 0.834, *p* = 0.004), emergency admission (OR: 1.192, *p* = 0.005), severe ipsilateral carotid stenosis (OR: 1.501, *p* = 0.022), and prior ipsilateral interventions (OR: 1.722, *p* = 0.003) emerged as significant factors that correlates with postoperative hypertension in unilateral surgeries. Conversely, in bilateral procedures, gender, emergency admissions (*p* = 0.012), and plaque morphology (*p* = 0.035) significantly influenced postoperative hypertension. Notably, 2.2% of bilateral surgery patients developed hyperperfusion syndrome, culminating in hemorrhagic stroke within 30 days. Intriguingly, postoperative stage II hypertension was identified as an independent predictor of neurological deficits post-secondary procedure in bilateral CEA cases (*p* = 0.004), attributable to hyperperfusion syndrome. However, it did not independently predict myocardial infarction or mortality outcomes. The overall 30-day stroke rate stood at 0.90%. Lowest incidence of post operative hypertension or any complications were observed in eversion carotid endartrertomy.

**Conclusion:**

The study identifies postoperative hypertension as a crucial independent predictor of perioperative stroke following bilateral carotid surgery. Moreover, the study elucidates the significant impact of bilateral CEA on the development of post-operative hyperperfusion syndrome or stroke, as compared to unilateral CEA. Currently almost 90% of our carotid practice is eversion carotid endartrerectomy.

## Introduction

Carotid endarterectomy (CEA) is a critical surgical intervention for the prevention of stroke in patients with carotid artery disease (1–3). However, this procedure is not without risks, particularly concerning hemodynamic instability. Approximately two-thirds of patients experience significant changes in blood pressure following CEA, primarily within the first 24 h (4). These fluctuations are pivotal as they directly influence perioperative morbidity, including stroke, myocardial infarction and death (5). Postoperative hypertension, reported in up to 56% of CEA patients, is especially concerning due to its potential to cause severe complications such as encephalopathy, cerebral haemorrhage, and cerebral hyperperfusion syndrome (CHS) (6, 7).

CHS is characterized by a substantial increase in cerebral blood flow (CBF), leading to symptoms of severe headaches, ipsilateral focal seizures, or intracerebral haemorrhage (8, 9). The clinical diagnosis of CHS can often be confirmed through non-contrast brain computed tomography (CT) scans or perfusion magnetic resonance imaging (MRI) (10).

A significant factor contributing to post-CEA blood pressure variations is the denervation of carotid sinus baroreceptors during the surgery (11, 12). This denervation, primarily due to the mobilization of the carotid bifurcation, disrupts the negative feedback mechanism controlling blood pressure, resulting in labile hypertension. While unilateral denervation typically leads to short-term blood pressure elevation, bilateral CEA can extend hemodynamic instability with hypertension, hypotension or bradycardia post-CEA for up to 12 weeks, posing a greater risk of its associated complications (12–14).

### Objectives

Our primary objective is to evaluate the incidence of cardiovascular hemodynamic instability in patients undergoing unilateral vs. bilateral carotid revascularization. This investigation aims to provide clear insights into the differential impacts of these two surgical approaches on neuro-cardiovascular stability.

## Methods

### Patient selection and data acquisition

This study encompasses a comprehensive retrospective analysis of patients who underwent open carotid surgery at our tertiary vascular referral centre from July 2002 to January 2023. During this time, we had 15,369 carotid referrals with more than 1,230 carotid interventions, of which 690 open carotid procedures were included who had complete medical records with ten years follow-up, including DUS follow-up and full neurological assessments. Amongst 690, 599 had unilateral, and 91 patients had bilateral carotid surgeries. During the follow-up, out of 599 unilateral carotid procedures, 60 patients developed recurrent stenosis, aneurysmal disease, and patch infection. Similarly, out of the 91 bilateral carotid procedures, eight patients developed the complications. In total, 43% (29 patients) of these patients were post Corona Virus Disease 2019 (COVID-19) infections. They were managed by trans carotid artery revascularization (TCAR) or interposition vein bypass graft and excluded from the analysis.

The data was meticulously sourced from Vascubase™ (Version 5.2, Consensus Medical Systems Inc., Richmond, BC), a database that collates patient information prospectively from clinical charts and administrative systems. Additional radiographic imaging data was extracted from our Picture Archiving and Communication System (PACS).

### Endpoints and definitions

The primary endpoints of this research are the incidence rates of postoperative hypertension, hyperperfusion syndrome and stroke following carotid revascularization surgery. Our investigation explores secondary endpoints: major adverse cardiovascular events and all-cause mortality. Major adverse cardiovascular events are stringently defined as any of the following occurrences: acute myocardial infarction (AMI), stroke, or cardiovascular mortality.

### Outcome measures and data analysis

To ensure precision and consistency in defining and measuring outcomes, we adhered to the Society for Vascular Surgery (SVS) Carotid Reporting Standards (15). The study records patients’ baseline demographics and vascular-related risk factors and comprehensively evaluates each patient's clinical presentation. This includes assessing the mode of admission, symptom status, the extent of ipsilateral and contralateral stenosis (duplex ultrasound scan (DUS) & computed tomography angiography (CTA)), plaque echo lucency (DUS), history of previous carotid surgery or neck radiation, perioperative cardiac medications, pre-procedural thrombolysis, and American Society of Anesthesiologists (ASA) physical status grade. This approach guarantees a thorough and authoritative analysis of the data.

### Surgical consideration

In this study, patients presenting symptoms within six months of onset were classified as symptomatic (1, 2). We strategically selected patients with an average surgical risk for operation. This included those with asymptomatic carotid stenosis ranging from 70% to 99% based on North American Symptomatic Carotid Endarterectomy Trial (NASCET) stenosis grading (16), particularly when associated with DUS imaging characteristics indicative of a heightened risk of late ipsilateral stroke, which included a ruptured hemorrhagic plaque, fresh echolucent material encasing calcific plaque, and ulceration with surface irregularity. Additionally, we operated on symptomatic patients exhibiting 50%–99% carotid stenosis (1, 16). However, we excluded elderly nursing home residents, sarcopenic patients, and cachexic patients with less than 2–3 years of life expectancy.

To ascertain the extent of carotid artery stenosis and evaluate plaque morphology DUS was employed. Following the SVS carotid reporting standards, carotid plaque morphology was categorized into distinct types: fibrous plaque/web, mild calcification, mixed fibrous/ulcerative plaques/thin cap, and multiple large calcification or lipid/necrotic cores (15). Echolucent plaques, characterized by a lipid-rich necrotic core, and echogenic plaques, identified by a fibrous-rich necrotic core, were also defined (15, 17). The vulnerable high-risk plaque included a ruptured hemorrhagic plaque, fresh echo lucent material encasing calcific plaque, and ulceration with surface irregularity. Diagnoses were corroborated using CTA and, or magnetic resonance angiography (MRA). CT scans were utilized to assess the extracranial and intracranial circulation, including the vertebral and carotid circulation and each segment of the circle of Willis.

### Procedure

CEA was performed under general anaesthesia, incorporating systemic heparinisation and prophylactic intravenous antibiotics. Notably, a carotid shunt was not employed, even in the absence of retrograde pulsatile flow from the unclamped ipsilateral internal carotid artery. We proactively managed systolic blood pressure (SBP), elevating it above 160 mmHg and adjusting it until cerebral oximetry readings exceeded 60%.

The arteriotomy closure techniques were diverse, including direct closure, a carotid patch application, or an eversion endarterectomy technique. The latter was selected in scenarios where the internal carotid artery (ICA) had a sufficient calibre (greater than 5 mm) and in cases necessitating the avoidance of a patch due to infection risks or where the ICA presented as coiled, kinked, or elongated. In eversion CEA, an oblique arteriotomy of the ICA at the carotid bulb was executed, facilitating the extirpation of the carotid plaque and the reimplantation of the ICA into the carotid bulb. All procedures entailed meticulous skeletonization of the carotid bulb post full clamping of proximal and distal inflow and outflow.

We prefer to wait for three months before embarking on the contralateral surgery to prevent laryngeal oedema and stridor. Furthermore, all our patients underwent a laryngoscopy to evaluate the vocal cord before the contralateral CEA to rule out possible asymptomatic homolateral inferior laryngeal nerve palsy (18). In these staged bilateral procedures, the initial surgery was categorized under the unilateral group, with the subsequent procedure classified as bilateral. It is crucial to note that there were no instances of simultaneous bilateral carotid surgery in this study.

### Follow-up protocol

In the postoperative phase, all patients were prescribed a dual antiplatelet regimen, aspirin and clopidogrel, for the first six months, followed by exclusive clopidogrel therapy thereafter. DUS was systematically conducted 6 weeks after the procedure and then at 6 month intervals over three years. Beyond this period, patients underwent annual DUS checks, extending up to ten years post-surgery.

### Outcomes definition

Postoperative hypertension was rigorously defined in line with the American Heart Association (AHA) guidelines, categorizing stage I hypertension as SBP ranging from 140 to 159 mmHg, and stage II hypertension as SBP ≥160 mmHg (19). Stroke, encompassing both disabling and non-disabling, ischemic or hemorrhagic types, was identified as any event occurring within 30 days post-procedure, ipsilaterally or contralaterally to the carotid surgery site.

All cases were reviewed by the vascular team, with diagnoses being validated through MRI or CT scans. AMI was diagnosed based on clinical presentation, electrocardiogram (ECG) changes, and abnormal troponin levels.

### Statistical analysis

Data was analyzed using IBM Statistical Package for the Social Sciences (SPSS) Version 22 (SPSS®, IBM® Corp., Armonk, N.Y., USA). The analysis encompassed descriptive statistics, employing means or medians for continuous variables and percentages or proportions for categorical variables. No data imputation was necessary given the minimal missing data (less than two percentages). Both univariate and multivariate analyses were utilized to identify factors associated with postoperative hypertension. Univariate regression analysis was applied to continuous and categorical data, calculating odds ratios where appropriate. Cox regression analysis was conducted on risk factors with significant univariate analysis *p*-values (<0.05). Kaplan–Meier survival estimates were used for time-to-event analysis, including restenosis, re-intervention, stroke, and death, with significance determined by a log-rank test.

### Ethical considerations

This study was conducted with full ethical approval from the Galway Clinical Research Ethics Committee (CA 1,687), ensuring adherence to the highest standards of research ethics and patient confidentiality.

## Results

The baseline demographics are given in Table 1.

**Table 1:** Comparison of baseline demographics, vascular-related risk factors and clinical presentation in unilateral and bilateral carotid interventions.

Preoperative risk factors	Unilateral (*N* = 599)	Bilateral (*N* = 91)	*p* value
Gender, male	66.11 (396)	73.00 (66)	0.216
Age, years	69.00 ± 8.49	69.23 ± 7.22	0.804
Age, >70 years	50.30 (301)	55.00 (50)	0.126
Smoker	34.72 (208)	32.00 (29)	0.309
Creatinine, µmol/L	94.95 ± 29.87	97.71 ± 34.29	0.425
Atrial fibrillation	13.00 (77)	12.10 (11)	0.703
Diabetes	20.00 (119)	20.00 (18)	0.613
Hypertension	74.00 (443)	79.12 (72)	0.501
Systolic blood pressure (pre-operative), mmHg	137.18 ± 20.04	138.76 ± 19.99	0.493
Diastolic blood pressure (pre-operative), mmHg	72.04 ± 10.86	72.32 ± 9.34	0.823
Hyperlipidemia	75.50 (452)	87.00 (79)	0.083
Ischemic heart disease	33.22 (199)	35.20 (32)	0.784
Heart failure	11.52 (69)	14.30 (13)	0.784
Chronic renal disease	10.52 (63)	14.30 (13)	0.655
Chronic lung disease	23.00 (137)	23.10 (21)	0.948
Emergency	32.00 (191)	27.50 (25)	0.322
Symptomatic	55.60 (333)	45.10 (41)	0.063
Ipsilateral stenosis, >70%	79.30 (475)	92.30 (84)	0.019
Contralateral stenosis, >70%	21.90 (131)	8.80 (8)	0.009
Plaque type, echolucent	14.52 (87)	14.30 (13)	0.049
Previous ipsilateral treatment	4.20 (25)	9.90 (9)	0.023
Previous neck radiation	0.50 (3)	0.0 (0)	–
Preoperative medication, ACEi^a^/ARB^b^	41.23 (247)	47.30 (43)	0.537
Preprocedural thrombolysis	24.70 (148)	22.00 (20)	0.557
ASA^c^ Grade, 4	3.20 (19)	3.30 (3)	0.812

^a^ ACEi, angiotensin-converting-enzyme inhibitors.

^b^ ARB, angiotensin receptor blockers.

^c^ ASA, American society of anesthesiologists.

Preoperatively, 74.00% (443/599) of patients undergoing unilateral and 79.12% (72/91) of patients undergoing bilateral carotid procedures were hypertensive.

Our protocol for the management of postoperative hypertension is to use glyceryl trinitrate (GTN) infusion if the patient has an ischemic heart, recent AMI or chronic obstructive pulmonary disease (COPD). Labelotol infusion is used for patients with normal cardiac and respiratory functions.

We excluded 68 patients (60 unilateral vs. 8 bilateral) from the outcome analysis. These patients had recurrent symptomatic carotid stenosis (*n* = 52, 37 unilateral vs. 15 bilateral), aneurysmal degradation of the patch (*n* = 9, 6 unilateral vs. 3 bilateral), and infection of the patch (*n* = 7, 2 unilateral vs. 5 bilateral), necessitating TCAR with flow reversal or excision of the patch with a bypass.

### Unilateral interventions

In unilateral carotid interventions, the mean SBP was 137.18 ± 20.04 preoperatively and 152.75 ± 25.72 postoperatively (*p* < 0.001). Postoperatively, 30.80% (*n* = 166) were normotensive, 26.00% (*n* = 140) had stage I hypertension, and 38.89% (*n* = 233) had stage II hypertension. Postoperatively, increased blood pressure was significantly greater in patients with contralateral carotid stenosis >90% (*p* = 0.002).

The risk factors of smoking (*p* = 0.007, OR: 1.183, 95%-CI: 1.040–1.345) and hyperfibrinogenemia (*p* = 0.004, OR: 0.834, 95%-CI: 0.738–0.941) were significantly associated with postoperative hypertension, respectively (Table 2).

**Table 2:** Comparison of baseline demographics, vascular-related risk factors and clinical presentation in normotensive and hypertensive patients after unilateral carotid surgery.

Preoperative risk factors	Normotensive (SBP < 140 mmHg) (*N* = 166)	Hypertensive (SBP ≥ 140 mmHg) (*N* = 373)	Odds ratio (OR)	*p*-value (95% confidence interval)
Gender, female	33.73 (56)	31.00 (115)	0.962	0.539 (0.850–1.090)
Age >80 years	9.03 (15)	8.60 (32)	1.020	0.846 (0.832–1.251)
Smoker	43.00 (71)	31.40 (117)	1.183	0.007 (1.040–1.345)
Hyperlipidemia	36.71 (61)	39.12 (146)	0.968	0.582 (0.863–1.086)
Diabetes	1.20 (2)	2.14 (8)	0.862	0.453 (0.629–1.181)
Hyperfibrinogenemia	27.11 (45)	37.53 (140)	0.834	0.004 (0.738–0.941)
Atrial fibrillation	14.00 (23)	13.14 (49)	1.020	0.819 (0.861–1.208)
Ischemic heart disease	16.00 (26)	13.70 (51)	1.050	0.558 (0.886–1.246)
Chronic kidney disease	3.01 (5)	4.60 (17)	0.890	0.396 (0.704–1.124)
Chronic lung disease	11.50 (19)	7.23 (27)	1.199	0.101 (0.934–1.538)
Emergency admission	54.00 (89)	67.00 (249)	1.192	0.005 (1.047–1.356)
Symptomatic	11.00 (18)	17.00 (63)	0.911	0.230 (0.790–1.051)
Plaque type, echolucent	25.30 (42)	24.00 (88)	1.251	0.304 (0.816–1.919)
Ipsilateral stenosis, >70%	76.00 (126)	82.04 (306)	0.875	0.085 (0.742–1.032)
Previous ipsilateral treatment	7.90 (13)	2.41 (9)	1.722	0.003 (1.039–2.854)
Contralateral stenosis, >70%	20.50 (34)	23.00 (85)	0.958	0.537 (0.841–1.093)
Previous contralateral treatment	3.61 (6)	2.70 (10)	1.111	0.555 (0.757–1.630)

In terms of clinical presentation, emergency admission (*p* = 0.005, OR: 1.192, 95%-CI: 1.047-1.356), ipsilateral carotid stenosis >90% (*p* = 0.022, OR: 1.501, 95%-CI: 1.059–2.129), previous ipsilateral carotid intervention (*p* = 0.003, OR: 1.722, 95%-CI: 1.039–2.854) were significantly associated with development of postoperative hypertension. However, the timing of surgery from the initial onset of symptoms (*p* = 0.729) was not associated with postoperative hypertension.

In terms of closure technique, 18.80% (*n* = 70) of CEA with primary closure and 14.50% (*n* = 31) with eversion endarterectomy procedures were associated with postoperative hypertension (*p* < 0.05) (Table 3).

**Table 3:** Surgical techniques and development of blood pressure (normotensive vs. hypertensive) after unilateral carotid surgery.

Surgical technique	Normotensive (SBP < 140 mmHg) (*N* = 166)	Hypertensive (SBP ≥ 140 mmHg) (*N* = 373)	*p*-value
Carotid endarterectomy with direct closure, % (*n*)	9.04 (15)	18.80 (70)	0.004
Carotid endarterectomy with patch plasty, % (*n*)	68.70 (114)	66.80 (249)	0.659
Eversion endarterectomy, % (*n*)	22.30 (37)	14.50 (54)	0.025

No incidence of CHS occurred in the unilateral group.

### Bilateral interventions

In bilateral carotid interventions, the mean SBP was 138.76 ± 19.99 mmHg preoperatively and 160.2 ± 25.0 mmHg postoperatively (*p* < 0.001). Postoperatively, 22.00% (*n* = 20) were normotensive, 22.00% (*n* = 20) had stage I hypertension, and 47.30% (*n* = 43) had stage II hypertension. The increase in blood pressure postoperatively was significantly greater in patients with contralateral recurrent carotid stenosis >90% (*p* < 0.001).

Demographics and vascular-related risk factors did not significantly influence the development of postoperative hypertension (Table 4). In terms of clinical presentation, female gender, emergency admission (*p* = 0.012) and plaque morphology (*p* = 0.035) were significantly associated with postoperative hypertension, respectively. Both were risk factors for CHS.

**Table 4:** Comparison of demographics, vascular-related risk factors, and clinical presentation in normotensive and hypertensive patients after bilateral carotid surgery.

Preoperative risk factors	SBP < 140 mmHg (*N* = 20)	SBP ≥ 140 mmHg (*N* = 63)	Odds ratio	*p*-value
Gender, female	25 (5)	27 (17)	1.025	0.861 (0.784–1.340)
Age >80 years	0 (0)	4.80 (3)	0.753	0.324 (0.665–0.853)
Smoker	45 (9)	52.40 (33)	0.962	0.748 (0.761–1.217)
Hyperlipidemia	90 (18)	89.00 (56)	1.057	0.763 (0.756–1.479)
Diabetes	30 (6)	19.04 (12)	1.182	0.285 (0.833–1.677)
Hyperfibrinogenemia	30 (6)	35.00 (22)	0.970	0.816 (0.750–1.254)
Atrial fibrillation	10 (2)	14.30 (9)	0.921	0.638 (0.677–1.253)
Ischemic heart disease	30 (6)	40.00 (25)	0.912	0.463 (0.721–1.155)
Chronic kidney disease	25 (5)	13.00 (8)	1.282	0.177 (0.820–2.003)
Chronic lung disease	25 (5)	27.00 (17)	0.976	0.861 (0.746–1.276)
Emergency admission	40 (8)	23.80 (15)	1.216	0.185 (0.878–1.685)
Symptomatic	10 (2)	3.20 (2)	1.371	0.455 (0.502–3.748)
Plaque type, echolucent	5 (1)	20.63 (13)	0.300	0.249 (0.035–2.574)
Ipsilateral stenosis, >70%	95 (19)	92.06 (58)	1.138	0.537 (0.819–1.580)
Previous ipsilateral treatment	10 (2)	9.52 (6)	1.018	0.934 (0.669–1.547)
Contralateral stenosis, >70%	15 (3)	7.93 (5)	1.237	0.351 (0.713–2.146)
Previous contralateral treatment	85 (17)	92.06 (58)	0.862	0.478 (0.535–1.390)

The timing of surgery from the initial onset of symptoms (*p* = 0.223) was not associated with postoperative hypertension. There was no significant association between the type of bilateral procedure and postoperative hypertension (*p* = 0.149).

In terms of closure technique, 17.50% (*n* = 11) with primary closure, 65.10% (*n* = 41) with patch, and 17.50% (*n* = 11) with eversion endarterectomy procedures were not significantly associated with postoperative hypertension (*p* = 0.313) (Table 5).

**Table 5:** Surgical techniques and development of blood pressure (normotensive vs. hypertensive) after bilateral carotid surgery.

Surgical technique	Normotensive (SBP < 140 mmHg) (*N* = 20)	Hypertensive (SBP ≥ 140 mmHg) (*N* = 63)	*p*-value
Carotid endarterectomy with direct closure, % (*n*)	15.00 (3)	17.50 (11)	0.794
Carotid endarterectomy with patch plasty, % (*n*)	55.00 (11)	65.10 (41)	0.417
Eversion endarterectomy, % (*n*)	30.00 (6)	17.50 (11)	0.226

### Post operative complications

Post-operative complications following unilateral carotid interventions are listed in Table 6, and bilateral carotid interventions in Table 7.

**Table 6:** Postoperative complications after unilateral carotid surgery.

Postoperative complications	Overall	Normotensive (*N* = 166)	Stage I hypertension (*N* = 140)	Stage II hypertension (*N* = 233)	*p*-value
Stroke <30 days	4	0.60 (1)	0 (0)	1.30 (3)	0.362
Overall stroke	11	1.80 (3)	2.14 (3)	2.14 (5)	0.968
Myocardial infarction	6	1.20 (2)	0.71 (1)	1.30 (3)	0.870
Mortality	151	29.52 (49)	19.30 (27)	32.20 (75)	0.024
Restenosis	71	12.70 (21)	12.90 (18)	13.73 (32)	0.871
Reintervention	5	1.80 (3)	0.71 (1)	0.43 (1)	0.358
Access site wound infection	4	0 (0)	2.90 (4)	0 (0)	–
Respiratory complications	24	4.80 (8)	3.60 (5)	4.72 (11)	0.845
Renal complications	1	0.60 (1)	0 (0)	0 (0)	–
Deep vein thrombosis	2	1.20 (2)	0 (0)	0 (0)	–
Pulmonary embolism	1	0 (0)	0 (0)	0.43 (1)	–
Procedural bleeding	8	0.60 (1)	0.71 (1)	2.60 (6)	0.185
Cervical hematoma	37	10.00 (16)	3.60 (5)	7.00 (16)	0.394
Cranial nerve injury	20	5.42 (9)	2.90 (4)	3.00 (7)	0.491
Access site pseudoaneurysm	2	0 (0)	0 (0)	0.90 (2)	–

**Table 7:** Postoperative complications after bilateral carotid surgery.

Postoperative complications	Overall	Normotensive (*N* = 20)	Hypertension (Stage I) (*N* = 20)	Hypertension (Stage II) (*N* = 43)	*p*-value
Stroke <30 days	2	0 (0)	0 (0)	4.5 (2)	–
Overall stroke	2	0 (0)	0 (0)	4.5 (2)	–
Myocardial infarction	2	0 (0)	0 (0)	4.8 (2)	–
Mortality	17	20 (4)	15 (3)	22.7 (10)	0.827
Restenosis	14	25 (5)	10 (2)	15.9 (7)	0.632
Wound infection	0	0 (0)	0 (0)	0 (0)	–
Respiratory complications	3	5 (1)	0 (0)	4.5 (2)	0.612
Renal complications	3	5 (1)	5 (1)	2.4 (1)	0.807
Deep vein thrombosis	0	0 (0)	0 (0)	0 (0)	–
Pulmonary embolism	0	0 (0)	0 (0)	0 (0)	–
Procedural bleeding	0	0 (0)	0 (0)	0 (0)	–
Cervical hematoma	1	0 (0)	5 (1)	0 (0)	–
Cranial nerve injury	2	0 (0)	0 (0)	8.3 (2)	–
Access site pseudoaneurysm	0	0 (0)	0 (0)	0 (0)	–

### Neurological complications

Overall, six patients developed a major ipsilateral stroke in the 30-day peri-procedural period (4 unilateral vs. 2 bilateral). Four of them were female patients. Three of the Reversible Ischeamic Neurological Deficit (RIND) occurred post-surgery for symptomatic carotid artery disease after discharge to the referral hospital. All recovered on medical treatment.

Of the patients who had unilateral carotid surgery, 0.74% (*n* = 4) had ishaemic stroke peri-operatively. Of these, 0.60% (*n* = 3) had stage II hypertension, and 0.20% (*n* = 1) was normotensive (*p* = 0.362) postoperatively.

Five patients developed amaurosis fugax in the 30-day periprocedural period, and one patient developed a TIA five weeks postoperatively, all of which had no sequelae. Of the five patients who developed amaurosis fugax, two were normotensive; one had stage I hypertension, and two had stage II hypertension postoperatively. All five patients had a unilateral carotid procedure. The patient who developed a postoperative TIA had a unilateral procedure and stage I hypertension postoperatively.

Of the patients who had bilateral carotid surgery, 2.20% (*n* = 2) developed a haemorrhagic stroke perioperatively due to CHS. These patients had stage II hypertension (*p* = 0.040).

Multivariate analysis found postoperative stage II hypertension to be an independent predictor of stroke (*p* = 0.004).

### Cardiovascular complications

In the 30-day peri-procedural period, 1.30% (*n* = 8) patients at our institution developed a non-fatal myocardial infarction; 1.11% (*n* = 6) of patients with unilateral carotid surgery and 2.40% (*n* = 2) with bilateral interventions developed a myocardial infarction postoperatively (*p* = 0.306).

Within the unilateral group, 1.30% (*n* = 3) patients with stage II hypertension, 0.71% (*n* = 1) patient with stage I hypertension and 1.20% (*n* = 2) patients who were normotensive developed a postoperative myocardial infarction (*p* = 0.870).

Within the bilateral group, both patients who developed a myocardial infarction postoperatively had stage II hypertension (*p* = 0.377).

Multivariate analysis did not find postoperative stage II hypertension to be an independent predictor of myocardial infarction (*p* = 0.475).

### Follow-up

The mean follow-up duration was 63.8 ± 41.7 months; 64.2 ± 41.3 months in unilateral groups vs. 61.3 ± 43.8 months in bilateral groups.

### All-cause mortality

**Figure 1:**
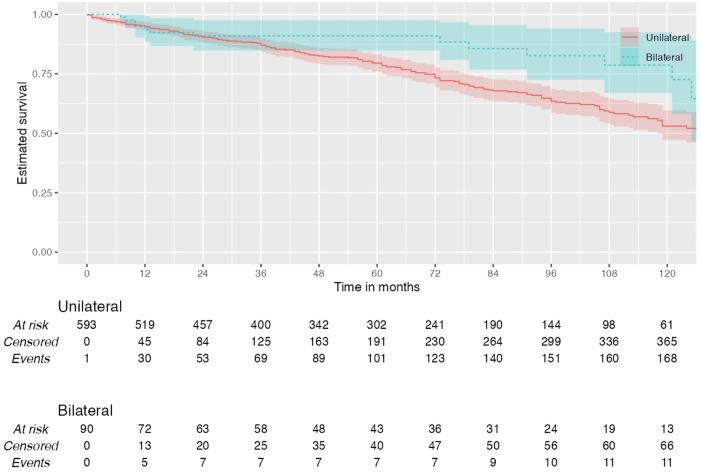
Kaplan–Meier plot showing all-cause mortality in patients with unilateral and bilateral carotid interventions.

During the total follow-up period of ten years, 31.83% (*n* = 198) of patients died. Among patients who had unilateral carotid surgery, 28.01% (*n* = 151) died postoperatively vs. 20.50% (*n* = 17) who had bilateral surgery (Log rank test: *χ*² 1.83**,** *p* = 0.176) (Figure 1).

Of all patients with unilateral surgeries who died postoperatively, 32.50% (*n* = 49) were normotensive, 17.90% (*n* = 27) had stage I hypertension, and 49.70% (*n* = 75) had stage II hypertension (*p* = 0.024). In these unilateral group, 10-year overall survival was 75.9% in normotensive, 85% in stage I hypertension and 74.5% in stage II hypertension.

Among patients who underwent bilateral carotid surgeries, **t**here was no significant association between postoperative blood pressure and mortality (*p* = 0.827). Of all patients with bilateral surgery who died postoperatively, 23.53% (*n* = 4) were normotensive, 17.65% (*n* = 3) had stage I hypertension, and 58.82% (*n* = 10) had stage II hypertension. In the bilateral group, 10-year overall survival was 71.4% in normotensive, 88.9% in stage I hypertension and 86.2% in stage II hypertension. Multivariate analysis did not find blood pressure to be an independent predictor of mortality (*p* = 0.608).

### Other post-operative complications

In the unilateral group, there were no significant associations between blood pressure and respiratory complications (*p* = 0.845), renal complications (*p* = 0.074), deep vein thrombosis (*p* = 0.107), pulmonary embolism (*p* = 0.517), procedural bleeding (*p* = 0.185), cervical hematoma (*p* = 0.394), cranial nerve injury (*p* = 0.491) and access site pseudoaneurysm (*p* = 0.267).

Similarly, in the bilateral group, there were no significant associations between blood pressure and respiratory complications (*p* = 0.612), renal complications (*p* = 0.807), cervical hematoma (*p* = 0.230) and cranial nerve injury (*p* = 0.310). There were no incidences of deep vein thrombosis, pulmonary embolism, procedural bleeding, wound infection, or access site pseudoaneurysm.

In terms of hemodynamic instability, acute hypotension occurred in 2.30% (*n* = 14) of patients’ post-operative, 2.23% (*n* = 12) in unilateral vs. 2.41% (*n* = 2) in bilateral groups (*p* = 0.177). Similarly, bradycardia occurred in 2.41% (*n* = 13) of patients, 2.23% (*n* = 12) unilateral vs. 1.20% (*n* = 1) bilateral. None of these patients developed a stroke or myocardial infarction. However, two patients in the unilateral group who had bradycardia died postoperatively (*p* = 0.736).

The need for re-intubation due to cervical oedema and stridor occurred in three patients in the bilateral carotid endarterectomy. The three patients had their second carotid surgery within 4 weeks. That led to a change of our practice for delayed extubation upto 24 hours post bilateral carotid endarterectomy.

## Discussion

In our comprehensive analysis, patients undergoing unilateral carotid surgery displayed comparable baseline demographics and risk factors to those undergoing bilateral interventions. However, significant variances were observed in aspects such as the degree of ipsilateral (*p* = 0.019) and contralateral stenosis (*p* = 0.009), plaque echo lucency (*p* = 0.049) and history of previous ipsilateral treatments (*p* = 0.023).

The prevalence of postoperative hypertension post-CEA, as reported in the literature, ranges broadly from 9% to 66% (6, 7). This variation could be attributed to divergent definitions of hypertension, inconsistencies in study designs, and varying follow-up durations (20). In our study, a notably high incidence of postoperative hypertension was observed—62.30% (*n* = 373) in unilateral (*p* < 0.001) and 69.23% (*n* = 63) in bilateral surgeries (*p* < 0.001). This could be linked to prevalent risk factors among our patients, including preoperative hypertension, diabetes mellitus, and significant carotid stenosis.

Our findings identified smoking (*p* = 0.729), hyperfibrinogenemia (*p* = 0.004) and previous ipsilateral carotid intervention (*p* = 0.003) as significant contributors to postoperative hypertension in unilateral surgeries. This contrasts with previous studies where age and diabetes were commonly associated with postoperative hypertension (21).

We observed that preoperative hypertension was highly prevalent in our patients, and it is a known factor for impaired baroreceptor physiologic reserve and further deterioration in baroreceptor sensitivity after carotid surgery (21–23). Following carotid surgery, a substantial proportion of these patients remained hypertensive, underlining the importance of preoperative blood pressure management.

Interestingly, while hypertension did not significantly correlate with perioperative stroke in unilateral surgeries (*p* = 0.362), it was a notable risk factor in staged bilateral procedures, as all the patients who developed perioperative stroke had stage II hypertension. This could be linked to increased mean SBP pre- and post-operatively in bilateral surgeries, highlighting the heightened risk of hyperperfusion syndrome and stroke due to baroreceptor dysfunction.

In patients with staged bilateral carotid procedures, we routinely wait for 6–12 weeks between the two interventions. Hypertension was significantly associated with the development of perioperative stroke (*p* = 0.040). Many studies have reported an association between postoperative hypertension and the development of stroke (21, 24). In our study, the difference in the significance of the association between hypertension and postoperative stroke in unilateral vs. bilateral carotid procedures may be attributed to a higher mean SBP pre- and post-operatively in patients who underwent bilateral surgery.

Studies have shown that patients undergoing bilateral carotid surgery have pre-existing baroreceptor dysfunction due to transection of the baroreceptor afferent nerve endings during the initial carotid surgery (25). Damage to another baroreceptor site with a bilateral carotid procedure can lead to a more severe hypertensive response, which increases stroke risk due to hyper-perfusion syndrome. The relationship between postoperative hypertension and neurological complications post-carotid surgery remains complex. While hypertension can be a contributory factor, other causes, like technical errors or thromboembolism, should be considered. Interestingly, four of the five patients who developed stroke postoperatively and had hypertension showed symptom resolution following medical management, suggesting the multifaceted nature of these complications.

Contrasting with the Asymptomatic Carotid Surgery Trial (ACST-1) (26), our study did not find a significant association between diastolic blood pressure and postoperative stroke or death in asymptomatic patients. This might be due to the one-time blood pressure measurement in ACST-1, highlighting the need for ongoing blood pressure monitoring.

Our study sheds light on the impact of high-grade ipsilateral carotid stenosis, which creates a state of chronic hypoperfusion, and the subsequent massive increase in CBF post-CEA. Interestingly, this was significantly associated with postoperative hypertension in bilateral procedures but not in unilateral ones (9, 27).

After removing a tight stenosis with CEA, impaired autoregulation mechanisms create a massive increase in CBF until eventual control is regained (9). Accordingly, our study found that ipsilateral high grade carotid artery stenosis was associated with postoperative hypertension in bilateral procedures.

Nouraei et al. (28) found that acute hemodynamic instability was significantly greater in patients with contralateral carotid stenosis. Chronic contralateral carotid stenosis impairs the baroreceptors that maintain blood pressure within the physiologic range and may cause a compensatory increase in the activity of the ipsilateral baroreceptor. Damage to this baroreceptor can lead to greater disturbance in blood pressure homeostasis after CEA in patients with contralateral carotid stenosis as the overall baroreflex buffering capacity is depleted. Accordingly, the results of our study showed that contralateral stenosis was associated with the development of postoperative hypertension after bilateral carotid surgery. However, postoperative CHS occurred in three patients, two females and one male; all were smokers, over 60 years of age, with symptomatic 99% stenosis and confirmed ruptured hemorrhagic plaques. All developed post opertative brain bleeds and were managed conservatively without any neurological sequelae.

Further, our findings align with previous research indicating that contralateral carotid stenosis can exacerbate postoperative hypertension by impairing baroreceptors (29). In our study, plaque echolucency was not associated with postoperative hypertension in unilateral (*p* = 0.304) and bilateral (*p* = 0.249) procedures, respectively. However, plaque echogenicity was significantly associated with postoperative stroke in bilateral surgeries, emphasizing the role of plaque morphology in postoperative complications (*p* = 0.039).

Emergency admissions due to acute symptom presentation were significantly associated with postoperative hypertension. This reinforces the challenge of managing blood pressure in patients requiring urgent surgery, especially considering the impaired baroreflex function in the initial weeks post-TIA or stroke. Recent evidence has emphasized the high incidence of disabling and non-disabling strokes occurring in the first few days after a TIA or stroke, thereby advocating urgent carotid revascularization. There is less time available to control for risk factors, including hypertension, in higher-risk patients presenting for urgent surgery. Cerebral oedema is a severe complication of acute ischemic stroke that increases intracranial pressure, which decreases cerebral perfusion and blood flow. A normal compensatory response to increased intracranial pressure is to increase blood pressure to maintain cerebral perfusion pressure (30). As baroreflex function is impaired the first few weeks after TIA or stroke, this could make perioperative arterial blood pressure management more difficult. In patients who previously experienced neurological symptoms (amaurosis fugax, TIA or a stroke) but were asymptomatic at the time of the procedure, the timing of surgery from the initial presentation was not significantly associated with postoperative hypertension after unilateral (*p* = 0.729) or bilateral (*p* = 0.223) carotid surgery. As preoperative hypertension was found to be significantly associated with postoperative hypertension, Naylor et al. found that deferring carotid surgery to optimize blood pressure control in asymptomatic patients may be an effective strategy to reduce complications due to postoperative hypertension and CHS (31).

Different surgical techniques, like eversion CEA, have been linked to varying degrees of hemodynamic instability. In our institution, the type of carotid procedure significantly influenced postoperative hypertension, especially in unilateral surgeries. This finding is crucial for surgical planning and patient management (11). An eversion CEA is performed through a transverse incision of the ICA at the carotid bulb (11, 22). The incision transects the carotid sinus nerve fibres that innervate the baroreceptors in the proximal ICA (22). Baroreceptor sensitivity falls following eversion CEA (32). Mehta et al. (22) found a four-fold increase in the use of intravenous vasodilators to control excessively elevated blood pressures after eversion CEA. At our institution, there was a significant association between the type of carotid procedure and postoperative hypertension after unilateral carotid surgery (*p* < 0.05). It was initially believed that acute changes in arterial pressure and heart rate during CEA were caused by manipulation of the carotid sinus (7). However, studies have found that the carotid atheroma reduces cerebral perfusion and impairs baroreceptor reflexes and cerebrovascular reactivity. Differences in carotid plaque morphology and echolucency between CEA with patch and eversion endarterectomy, as well as other theories, including alterations in the renin-angiotensin system, vasopressin concentrations or central catecholaminergic activity, may explain the higher rate of perioperative hypertension in CEA with patch procedures (7). An eversion CEA has technical procedural advantages compared to a CEA with a patch that may also explain a lower incidence of postoperative hypertension after this procedure in our study. These advantages include shortening a redundant ICA and faster anastomosis of the ICA to the carotid bulb.

Wong et al. (33) found a significant association between postoperative hypertension with stroke or death (*p* = 0.04) and cardiac complications (*p* = 0.07). Hirschl et al. (34) found that in patients undergoing carotid endarterectomy, the occurrence of blood pressure instability was associated with a 3.3 times higher, statistically significant risk of developing major cardiovascular complications and an eightfold increased risk of cardiovascular mortality in the five years after the operation. Towne and Bernhard found a significantly increased incidence of neurological deficit and operative mortality in patients who developed postoperative hypertension (24). In our study, multivariate analysis found blood pressure to be an independent predictor of stroke (*p* = 0.004). There was no significant association between postoperative hypertension and myocardial infarction or death after unilateral and bilateral carotid surgery.

Randomized clinical trials do not have data comparing the optimal perioperative management protocol for patients with hypertension and CHS (10). Intensive hemodynamic monitoring of systemic blood pressure and heart rate postoperatively in high-risk patients, prompt diagnosis and initiation of appropriate treatment is the cornerstone of prevention and management. There are no definitive criteria for the level of systolic blood pressure to initiate therapy and choice of antihypertensive drug. In a 21-year audit of strategies used for prevention and stroke following CEA, Naylor et al. developed guidelines for the management of hypertension (>170 mmHg in theatre and >160 mmHg on the wards) (25). According to these guidelines, the first-line pharmacologic agent is labetalol, with hydralazine as the second line and glyceryl trinitrate as the third-line (31, 35). If patients who are not on antihypertensive therapy become hypertensive on the ward in the absence of headaches or other neurologic deficits, nifedipine is the first line, followed by bisoprolol and then ramipril. If the patient is on antihypertensive therapy, the choice of pharmacologic agent is determined by which agent(s) the patient is currently taking. We use GTN for patients with ischeamic heart disease and labetalol for all other patients.

In some studies, proactive use of atropine was beneficial for bradycardia, and beta-blockers were discontinued preoperatively to decrease baroreceptor stimulation-related bradycardia and hypotension (36). A systematic review and meta-analysis on the role of local anaesthetic blockage of the carotid sinus in reducing postoperative blood pressure following carotid surgery found insufficient data to draw any conclusions and exclude the possibility of harm (37). At our centre, we always instil local anaesthetic preoperatively and surround the carotid bulb with carotid sinus nerve blockade. In patients with CHS, measures to control cerebral oedema (sedation, hyperventilation, intravenous administration of glycerol or mannitol) and seizures (anticonvulsant therapy) are necessary (27, 38).

The incidence of postoperative hypotension has been reported in 12%–40% of CEA (33, 39, 40). An association between hypotension and perioperative morbidity remains unclear (20). Some studies report increased mortality and MI in hypotensive patients (39). Other studies found hypotension to be a transient and benign condition (40). In our study, clinically significant hypotension was defined as using intravenous medication to control hypotension. Amongst patients who underwent unilateral carotid surgery, 2.23% (*n* = 12) developed postoperative hypotension vs. 2.41% (*n* = 2) of patients who underwent bilateral interventions. Postoperative complications were not associated with hypotension after unilateral or bilateral carotid surgery. We believe that intensive care with strict BP control with mean arterial pressure of 75–95 mmHg and systolic above 120 and below 160 mmHg postoperatively is crucial in the first 24 h for unilateral patients and 48–72 h for bilateral patients. This algorithm could prevent post-CEA hyperperfusion and CHS (41–43).

### What we learned

While our study did not find a direct association between postoperative hypertension and myocardial infarction or mortality, the importance of intensive hemodynamic monitoring and timely intervention in high-risk patients cannot be overstated. The choice of antihypertensive medication, particularly in the context of patients’ existing medication regimens, is critical in managing these complications (41–43).

Our findings underscore the complexity of managing postoperative hypertension in carotid surgery patients. They highlight the need for personalized treatment plans, considering patient-specific risk factors, surgical techniques, and the nuanced relationship between blood pressure and postoperative complications. These insights will significantly influence our future clinical practice, emphasizing proactive, patient-centered care to mitigate risks associated with carotid surgeries.

Our study delineates several pivotal findings with far-reaching implications for the management of carotid surgery. Firstly, postoperative hypertension emerges as a significant independent predictor of perioperative stroke following bilateral carotid surgery. This underscores the need for vigilant blood pressure monitoring and management in the postoperative phase, particularly in patients undergoing bilateral procedures.

We identified hyperfibrinogenemia and a history of previous ipsilateral treatment as notable risk factors for postoperative hypertension. This insight should inform preoperative assessments, enabling clinicians to stratify patients based on their individual risk profiles and tailor perioperative care accordingly.

Contrary to previous research, our study revealed that the timing of surgery relative to the onset of neurological symptoms did not significantly impact the development of postoperative hypertension. These findings challenge existing paradigms and suggest that other factors, perhaps more nuanced and patient-specific, play a critical role in postoperative blood pressure changes (40–43).

In the context of surgical techniques, bilateral patch closure CEA was associated with the highest risk of postoperative hypertension and CHS vs. Bilareral eversion CEA. This highlights the necessity for surgical teams to carefully consider the choice of technique based on patient-specific risk factors and the potential for postoperative complications.

A key learning point from our research is the potential benefit of deferring elective carotid surgery in asymptomatic patients with high-risk plaques. By allowing time for optimal blood pressure control, we can significantly reduce the risks associated with postoperative hypertension and CHS. This strategy advocates for a more cautious and patient-centric approach, especially in managing asymptomatic high-risk individuals (43).

### Study limitations

One of the limitations of our study is its retrospective nature. Furthermore, bilateral CEAs are uncommon compared to unilateral CEA. However, we are witnessing more bilateral CEA due to COVID-19 infection. Given the systematic nature of atherosclerosis and good follow-up, we expect to encounter more cases of bilateral CEAs in the future.

## Conclusion

Our findings confirm that bilateral CEA influences the development of postoperative hypertension more markedly than unilateral procedures. This distinction is crucial for preoperative planning and counselling, emphasizing the need for enhanced vigilance and tailored management strategies in patients undergoing bilateral surgeries. Our study provides valuable insights and learning points that will profoundly impact future clinical practice. It emphasizes the importance of individualized patient assessment, meticulous surgical planning, and proactive postoperative management to mitigate the risks associated with carotid surgeries.

## Data Availability

The raw data supporting the conclusions of this article will be made available by the authors, without undue reservation.
